# Population compliance with COVID-19 directions in December 2021, Queensland, Australia

**DOI:** 10.5365/wpsar.2023.14.4.1018

**Published:** 2023-11-29

**Authors:** Marguerite Dalmau, Ramim Sourjah, Ross Andrews, Emma Field, Stephen Lambert

**Affiliations:** a National Centre for Epidemiology and Population Health, The Australian National University, Canberra, Australia.; b Communicable Diseases Branch, Queensland Health, Brisbane, Queensland, Australia.; c COVID-19 Public Health Response Division, Queensland Health, Brisbane, Queensland, Australia.; d National Centre for Immunisation Research and Surveillance, Westmead, New South Wales, Australia.

To contain the spread of coronavirus disease (COVID-19), most countries introduced travel-related control measures such as restricted entry, quarantine of travellers and screening requirements. (*1*) The Australian response to the COVID-19 pandemic included the closure of the international border and restricted movement between Australian states and territories. In the state of Queensland, *Border Restrictions Direction (No. 56)* came into effect on 13 December 2021. The direction, enacted by the state’s Chief Health Officer, stipulated that a person entering Queensland from a declared national COVID-19 hotspot must meet the following conditions: not be an international arrival, be fully vaccinated (≥ 2 doses), have received a negative COVID-19 polymerase chain reaction (PCR) test result within 72 hours before arrival, and undertake a COVID-19 PCR test on day 5, or as close to day 5 as practical, after arrival. (*2*) People entering Queensland were required to complete an electronic border pass application within 72 hours before their arrival, declaring their vaccination status and willingness to comply with the order. There are limited data in the public domain reporting compliance with public health directions during the COVID-19 pandemic in Australia. This report summarizes the findings of an audit activity to determine compliance with the Queensland directive that came into force in mid-December 2021 and to assess whether the process could be scaled up for continued compliance monitoring.

Queensland border pass applications lodged between midnight on 12 December 2021 and 21:48 on 13 December 2021 were analysed (data extraction: 16 December 2021). Eligibility required the applicant to be a returning or new Queensland resident with a Queensland residential address recorded. Automatic data linkage methods were used to connect extracted border pass application data, COVID-19 vaccination status in the Australian Immunization Register (AIR), and evidence of a COVID-19 test from the Queensland Notifiable Conditions System (NoCS). To evaluate process completeness, manual linkage checks were performed for unmatched records. This analysis was conducted as a public health audit activity in accordance with the Queensland *Public Health Act 2005*. (*3*) Data were extracted and stored on password-protected organizational devices with auditable, individual-user monitoring capabilities. Raw data were available only to staff directly involved in the data linkage. Data were de-identified for analysis and then aggregated for reporting.

**Fig. 1:**
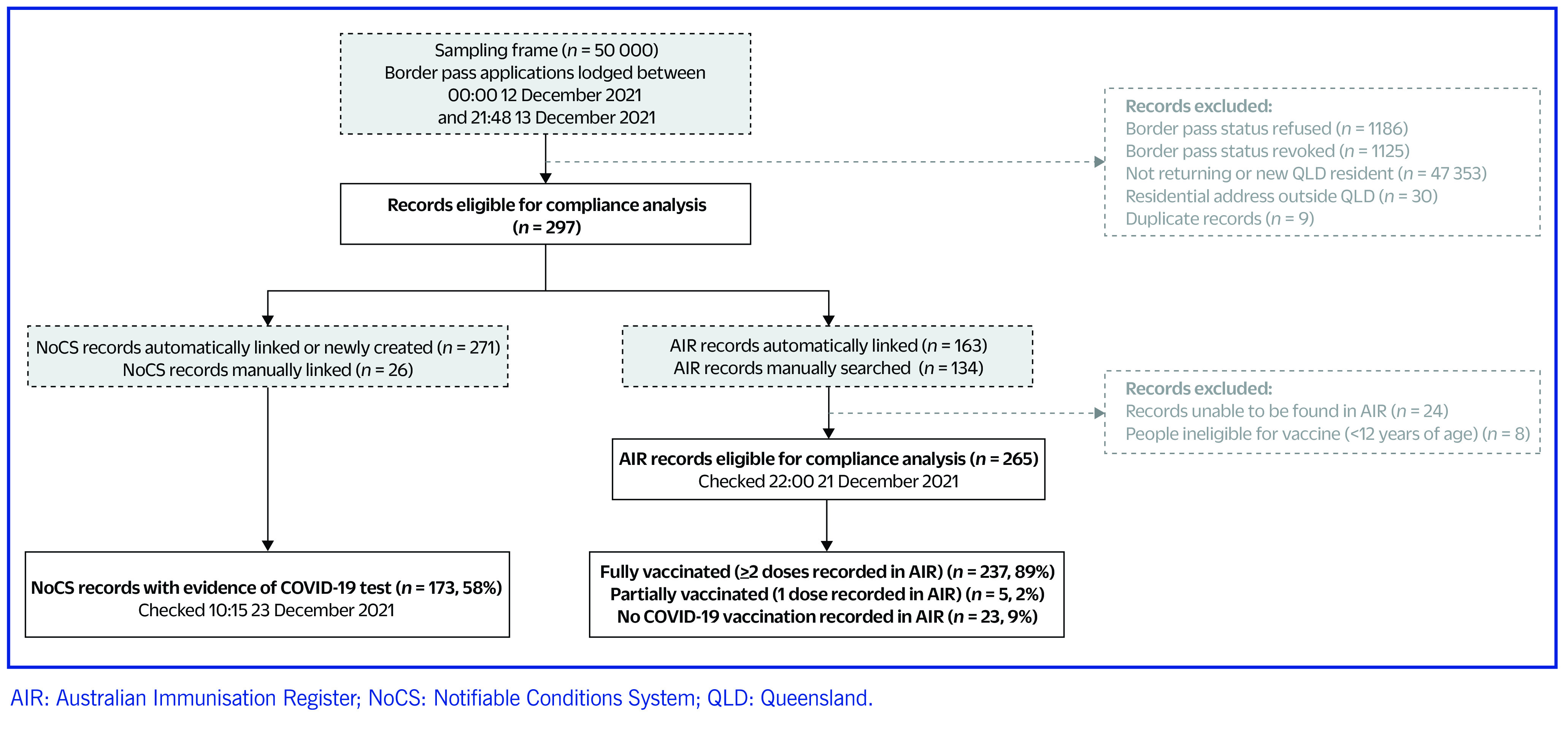
Summary of compliance analysis process and results

From the eligible sample of new and returning Queensland residents (*n* = 297), 173 (58%) were matched to a COVID-19 test in NoCS in the 9-day period after 13 December 2021 (**Fig. 1**). Of these, 26 (9%) NoCS records were identified via manual searching. The automatic linkage process with the AIR successfully matched 163 (55%) records, while the remaining 134 (45%) were manually searched. Of the 265 residents whose vaccination status could be viewed in the AIR and were eligible for vaccination, 237 (89%) were identified as fully vaccinated.

[**insert** Figure 1]

Policy responses to the COVID-19 pandemic have been dynamic, and public health directions have changed frequently in line with emerging evidence. (*4*) In Australia, information on population compliance with these directions has been limited, more commonly measured through the identification of individual breaches. Our analysis provides a cross-sectional estimate of vaccination and testing compliance among new and returning Queensland residents in December 2021. The high vaccination compliance (89%) reflected broader community vaccination coverage. However, compliance with post-arrival PCR testing was lower, possibly because of the burden on the health system at the time, which resulted in long testing wait times and delayed results.

One limitation of our analysis was the completeness and availability of the data from the border pass applications system. The supplied data did not list date of entry to Queensland. People were required to lodge their applications within 72 hours of entry, resulting in a broad compliance indication instead of a true “day 5” testing representation. The process could match 58% of people to a test recorded in NoCS in the 9-day period after 13 December 2021. Having accurate entry dates could have provided a more accurate population sample and resulting day 5 compliance value. Another limitation was the role of residential address in automated COVID-19 test reporting. Due to the processing of test results based on residential address, testing compliance through NoCS could only be reliably assessed for people with Queensland residential addresses. This resulted in an incomplete sample and limited our understanding of compliance by all entrants to the state, at a time when border restrictions were being eased and many visitors were entering the state.

Although we could estimate compliance for those with Queensland residential addresses, we could not include people entering Queensland without a Queensland residential address. Also, manual input was required to assess vaccination status, with 45% of vaccination records needing to be manually matched. These issues would create barriers to population-level scale-up of this audit activity. Thus, further consideration is required to improve connectivity and integration of public health information management systems. In future, it will be important to consider system capacity to monitor compliance with public health directions during health emergencies, and to improve communication and engagement mechanisms when non-compliance occurs. Ideally, future work could include an extended period of analysis to strengthen our understanding of compliance over time. Although monitoring compliance is a valuable element of an emergency public health response, it is not always feasible within existing infrastructure or resources of the health system. Communication and engagement mechanisms before an emergency response or before implementation of public health measures could strengthen compliance and reduce the need for monitoring.
